# Short-term lipopolysaccharide treatment leads to astrocyte activation in LRRK2 G2019S knock-in mice without loss of dopaminergic neurons

**DOI:** 10.21203/rs.3.rs-4076333/v1

**Published:** 2024-03-21

**Authors:** Hoang Kieu Chi Ngo, Hoang Le, Samuel J. Ayer, Grace F. Crotty, Michael A. Schwarzschild, Rachit Bakshi

**Affiliations:** Massachusetts General Hospital; Massachusetts General Hospital; Massachusetts General Hospital; Massachusetts General Hospital; Massachusetts General Hospital; Massachusetts General Hospital

**Keywords:** Parkinson’s disease, dopaminergic neuronal loss, LRRK2, G2019S, autophosphorylaton, LPS, neuroinflammation, astrocyte activation, GFAP, caffeine

## Abstract

**Background:**

The G2019S mutation of LRRK2, which enhances kinase activity of the protein, confers a substantial risk of developing Parkinson’s disease (PD). However, the mutation demonstrates incomplete penetrance, suggesting the involvement of other genetic or environmental modulating factors. Here, we investigated whether LRRK2 G2019S knock-in (KI) mice treated with the inflammogen lipopolysaccharide (LPS) could model LRRK2 PD.

**Results:**

We found that short-term (2 weeks) treatment with LPS did not result in the loss of dopaminergic neurons in either LRRK2 G2019S KI or wild-type (WT) mice. Compared with WT mice, LRRK2 G2019S-KI mice showed incomplete recovery from LPS-induced weight loss. In LRRK2 G2019S KI mice, LPS treatment led to upregulated phosphorylation of LRRK2 at the autophosphorylation site Serine 1292, which is known as a direct readout of LRRK2 kinase activity. LPS treatment caused a greater increase in the activated astrocyte marker glial fibrillary acidic protein (GFAP) in the striatum and substantia nigra of LRRK2 G2019S mice than in those of WT mice. The administration of caffeine, which was recently identified as a biomarker of resistance to developing PD in individuals with *LRRK2* mutations, attenuated LPS-induced astrocyte activation specifically in LRRK2 G2019S KI mice.

**Conclusions:**

Our findings suggest that 2 weeks of exposure to LPS is not sufficient to cause dopaminergic neuronal loss in LRRK2 G2019S KI mice but rather results in increased astrocyte activation, which can be ameliorated by caffeine.

## Background

Parkinson’s disease (PD) is a progressive condition caused by the degeneration of dopaminergic neurons in the substantia nigra pars compacta and the formation of α-synuclein-containing Lewy bodies in surviving neurons(1). The majority of PD cases are sporadic or idiopathic; however, an increasing number of genes are reportedly associated with familial forms of the disease(1, 2). Mutations in the leucine-rich repeat kinase 2 (*LRRK2*) gene are considered the most frequent genetic causes of PD(3), making LRRK2 a potential therapeutic target for PD(4, 5).

G2019S is one of the most common mutations among a dozen different LRRK2 mutations reported in PD(5). It has been found in approximately 5–7% of familial (autosomal dominant) and 1–2% of sporadic PD patients(6). The G2019S mutation in LRRK2 confers a toxic gain of function, likely via increased LRRK2 kinase activity(7). This increase in LRRK2 kinase activity has been strongly implicated in PD pathogenesis(6, 8, 9). However, the LRRK2 G2019S mutation demonstrates incomplete penetrance, even in the homozygous state(5). Neither LRRK2 G2019S transgenic (Tg) nor LRRK2 knock-in (KI) mice fully recapitulate human PD, including age-dependent degeneration of dopaminergic neurons and α-synucleinopathies(10, 11). This finding suggested that other genetic or environmental modulating factors are required for the LRRK2 G2019S mutation to trigger dopaminergic neuronal loss(12).

LRRK2 is highly expressed in glial cells, which play major roles in neuroinflammation(13, 14). Neuroinflammation is triggered by brain trauma or exposure of the nervous system to toxins or infections and is regulated by local components of the immune system in the central nervous system (CNS) and peripheral immune cells recruited to the CNS(15). An acute neuroinflammatory response is essential for clearing pathogens and prompt repair of damaged tissues. However, when neuroinflammation is not resolved, neuroinflammation becomes chronic and can be detrimental to neurons. Under such conditions, glial cells are activated and release proinflammatory and neurotoxic factors that induce neuronal damage and neurodegeneration(15, 16). Activated glial cells, including astrocytes and microglia, have been reported in preclinical models of PD (17) and PD brains(18). The inhibition of glial activation is neuroprotective in murine PD models, indicating the importance of neuroinflammation in PD pathogenesis(19). Injection of the gram-negative bacterial endotoxin lipopolysaccharide (LPS) was shown to be efficient in causing inflammatory dopaminergic neurodegeneration(20). It was also reported that LRRK2 G2019S Tg mice but not wild-type (WT) or LRRK2 Tg mice displayed dopaminergic neuronal loss upon long-term exposure to a single high dose of LPS(21). Neuronal loss occurred seven days after LPS injection and then stabilized(21). Thus, we wanted to adapt this paradigm to test whether we can observe similar results in LRRK2 G2019S KI mice with murine LRRK2 G2019S mutant protein expressed at a physiological level(22).

In the search for biomarkers of resistance to developing PD in individuals with and without *LRRK2* mutations, our group identified caffeine and its related metabolites as potential modulators(23). Plasma concentrations of caffeine in participants with PD were lower than those in unaffected controls, even more so among *LRRK2* carriers with PD than among their control counterparts(23). The metabolomics findings suggest that caffeine could have neuroprotective effects that are specific to *LRRK2* mutation carriers. We hypothesized that if caffeine confers protection against *LRRK2* PD, then this effect might be attributable to caffeine’s potential to modulate neuroinflammation.

In this study, by exposing WT and LRRK2 G2019S KI mice to a single sublethal dose of LPS, we showed that the G2019S mutation of LRRK2 did not lead to the loss of dopaminergic neurons two weeks after LPS treatment but caused a delay in recovery from LPS-induced weight loss. We also found that LPS treatment led to increased LRRK2 autophosphorylation at Ser 1292 in LRRK2 G2019S KI mice. Compared with their WT counterparts, LRRK2 G2019S-KI mice displayed enhanced LPS-triggered astrocyte activation, and caffeine attenuated this astrocyte activation specifically in LRRK2 G2019S-KI mice challenged with LPS. Our findings suggest that the LRRK2 G2019S mutation contributes to astrocyte activation and that caffeine could be a potential therapeutic candidate for LRRK2 PD.

## Methods

### Animals

Animal care and husbandry were performed according to the Massachusetts General Hospital (MGH) Subcommittee on Research Animal Care guidelines. All animal procedures were approved by the MGH Institutional Animal Care and Use Committee under the National Institutes of Health’s guidelines for the Care and Use of Laboratory Animals (24) (approval number: 2006N000120). The experiments were rigorously conducted following the ARRIVE guidelines (25) by researchers who were blinded to the genotypes and treatments.

#### LRRK2 G2019S KI mice.

LRRK2 G2019S KI mice (B6.Cg-Lrrk2tm1.1Hlme/J) were obtained from the Jackson Laboratory (USA). Age-matched male WT and LRRK2 G2019S-KI mice were used in this study. Genotyping was performed from ear clips using the following primers: forward primer, 5 - CAC CCC AGG TAG GAG AAC AA-3 ; reverse primer, 5 - TGC CAT GGT CAT TAC TCT TCA-3 (22). Briefly, earpieces were incubated in genotyping buffer containing 1% sodium dodecyl sulfate (SDS; Cat. #AM9822; Thermo Fisher Scientific, USA), 0.1 mM sodium chloride (NaCl; Cat. #AM9760G; Thermo Fisher Scientific, USA), 100 mM EDTA (Cat. #AM9260G; Thermo Fisher Scientific, USA), 50 mM Tris (pH 8.0; Cat. #15568025; Thermo Fisher Scientific, USA), and 1 mg/mL proteinase K (Cat. #EO0491; Thermo Fisher Scientific, USA) at 55°C overnight. On the following day, the same volume of 5 mM NaCl was added to the homogenates to pellet the cell debris. The mixture was then centrifuged at 13,000 × *g* for 15 minutes at 4°C. DNA-containing supernatants were carefully collected, followed by incubation with cold isopropanol (Cat. #BP2618500; Thermo Fisher Scientific, USA) for 2 hours (h) at − 20°C to precipitate the DNA. DNA pellets were washed with cold 70% ethanol (Cat. #BP2818100; Thermo Fisher Scientific, USA), dried, and resuspended in pure DNase/RNase-free distilled water (Cat. #10977015; Thermo Fisher Scientific, USA). A standard PCR procedure was performed with three hundred nanograms of genomic DNA used as a template. PCR products were separated on 2% ethidium bromide (EtBr; Cat. #BP1302–10; Thermo Fisher Scientific, USA)-stained agarose gels (Cat. #50004; Lonza, USA) and visualized under a ChemiDoc MP Imaging System (Bio-Rad, USA). The WT and LRRK2 G2019S mutant alleles generated 130 bp bands and 223 bp bands, respectively, on the gels.

### LPS treatment

Eight-month-old male mice were given a dose of 5 mg/kg LPS (Escherichia coli serotype O111:B4; Cat. #L2630; Sigma–Aldrich, USA) prepared in 0.9% saline (Cat. #S5819; Teknova, USA) via intraperitoneal (i.p.) injection(21). Body weights were monitored daily for two weeks.

### Brain tissue collection

Two weeks post-LPS injection, the mice were sacrificed by CO_2_ asphyxiation and then perfused with 0.9% saline. The striatum from each hemisphere was isolated, snap-frozen on dry ice, and stored at − 80°C until use. The left striatum was used for measuring striatal levels of dopamine and its metabolite 3,4-dihydroxyphenylacetic acid (DOPAC). The right striatum was subjected to Western blot analysis to measure the expression levels of proteins of interest. Blocks of tissue containing the substantia nigra were dissected and fixed in 4% paraformaldehyde (PFA; Cat. #15714-S; Electron Microscopy Sciences, USA) in phosphate-buffered saline (PBS; pH 7.4; Cat. #P2100–050; GenDepot, USA) until further analyses.

### Neurochemical analysis

The frozen striata were weighed and homogenized in a solution consisting of 0.1 M phosphoric acid (Cat. #345245; Sigma–Aldrich, USA), 0.1 mM EDTA, and 100 ng/mL 3,4-dihydroxybenzylamine (DHBA; Cat. #858781; Sigma–Aldrich, USA) as an internal standard at a 1:20 (weight:volume) ratio. The crude homogenates were cleared by centrifugation at 10,000 × *g* for 15 minutes at 4°C, and the supernatants were carefully collected. After microfiltration of the supernatants through Costar Spin-X 0.22 μm centrifuge tube filters (Cat. #CLS8160; Sigma–Aldrich, USA), the levels of dopamine and its primary metabolite DOPAC in the filtrates were analyzed using a high-performance liquid chromatography-electrochemical detection (HPLC-ECD) system (Cat. #Ultimate 3000 UHPLC; Thermo Fisher Scientific, USA). The separation was performed on a Microsorb-MV column (C18, 150 × 4.6 mm, C18, 5 μm; Cat. # AG-R0089200D5; Agilent Technologies, USA) at a flow rate of 0.6 mL/min with a mobile phase consisting of 75 mM sodium phosphate monobasic (NaH_2_PO_4_; Cat. #71504; Sigma Aldrich, USA), 1.7 mM sodium 1-octanesulfonate (Cat. #74885; Sigma–Aldrich, USA), 100 μL/L triethylamine (Cat. #47128; Sigma–Aldrich, USA), 25 μM EDTA, and 10% (v/v) acetonitrile (Cat. #AA22927M1; Thermo Fisher Scientific, USA). The autosampler was set at 4°C, and the injection volume was 10 μL. Detection was achieved with an electrochemical detection system (Cat. #Ultimate 3000 ECD-3000RS; Thermo Fisher Scientific, USA) with screening and detection electrodes set to − 150 mV and 250 mV, respectively. Dopamine (Cat. #73483; Sigma–Aldrich, USA) were used as standards. The concentrations of the analytes were calculated from the corresponding standard curves.

### Western blot analysis

Striata were homogenized in RIPA lysis and extraction buffer (Cat. #89900; Thermo Fisher Scientific, USA) supplemented with a protease inhibitor cocktail (Cat. #78429; Thermo Fisher Scientific, USA). The concentrations of proteins in the lysates were quantified by a bicinchoninic acid (BCA) protein assay kit (Cat. #PI23225; Thermo Fisher Scientific, USA). Total proteins were separated by sodium dodecyl sulfate–polyacrylamide gel electrophoresis (SDS–PAGE; NuPAGE gels; Cat. #NP0323BOX; Thermo Fisher Scientific, USA). High-molecular-weight proteins such as LRRK2 were separated on 3–8% Tris-acetate gels (Cat. #EC66255BOX; Thermo Fisher Scientific, USA). The proteins were transferred to nitrocellulose membranes (Cat. #10-6000-09; GE Healthcare Life Sciences, USA). Membranes were then blocked with a solution of 5% skim milk (Cat. #232100; BD Life Sciences, USA) prepared in 0.1% PBST (14) for 1 h at room temperature (RT). This was then followed by incubation with the indicated primary antibodies diluted in PBST at 4°C overnight. The primary antibodies used were as follows: rabbit monoclonal anti-LRRK2 (1:2000; Cat. #ab133474; Abcam, USA), rabbit monoclonal anti-P-LRRK2 (Ser 1292; 1:1000; Cat. #ab203181; Abcam, USA), rabbit RAB12 polyclonal antibody (1:2000; Cat. #PA5–48179; Invitrogen, USA), rabbit anti-RAB12 (Ser 106; 1:1000; Cat. #ab256487; Abcam, USA), rat monoclonal anti-glial fibrillary acidic protein (GFAP; 1:2000; Cat #13–0300; Invitrogen, USA), goat polyclonal anti-ionized calcium-binding adaptor molecule 1 (Iba1; 1:1000; Cat. #ab5076; Abcam, USA), rabbit polyclonal anti-tyrosine hydroxylase (TH; 1:2000; Cat. #BML-SA497–0100; Enzo Life Sciences, Inc., USA), mouse monoclonal anti-α-tubulin (1:2000; Cat. #T9026; Millipore Sigma, USA), and mouse monoclonal anti-glyceraldehyde-3-phosphate dehydrogenase (GAPDH; 1:2000; Cat. #sc-365062; Santa The following day, after three washes in 0.1% PBST, the membranes were treated with corresponding horseradish peroxidase (HRP)-conjugated secondary antibodies (goat anti-mouse secondary antibody, HRP: Cat. #31430; goat anti-rabbit secondary antibody, HRP: Cat. #31460; goat anti-rat secondary antibody, HRP: Cat. #31470; Invitrogen, USA) prepared in 0.1% PBST containing 2.5% skim milk for 1 h at RT. The blots were washed again with PBST three times and incubated with an enhanced chemiluminescent (ECL) substrate (Cat. #34094; Thermo Fisher Scientific, USA) before being imaged with an Odyssey^®^ XF Imaging System (LI-COR Biosciences, USA). The intensities of the protein bands were measured by ImageJ (NIH) and normalized to those of the corresponding loading controls.

### Immunohistochemical analysis

PFA-fixed substantia nigra-containing brain blocks were dehydrated in 30% sucrose (Cat. #15-503-022; Invitrogen, USA) in PBS (pH 7.4) for 48 h. Brains were serially sectioned into 30-μm slices in the coronal plane using a microtome (Cat. #SM 2010; Leica, USA). Sixth sections of the entire midbrain were used for staining. Brain slices were washed three times (7 minutes each) with PBS (pH 7.4) and incubated with 3% H_2_O_2_ (Cat. #H325–500; Fisher Scientific, USA) in PBS (pH 7.4) for 12 minutes to quench endogenous peroxidases. The sections were subsequently washed three times (7 minutes each) with PBS (pH 7.4) and blocked with blocking buffer containing 0.3% Triton X-100 (Cat. #11332481001; Sigma Aldrich, USA) and 5% (v/v) normal goat serum (Cat. #S-1000–20; Vector Laboratories, USA). The sections were then incubated with a primary rabbit polyclonal antibody against TH (1:500; Cat. #BML-SA497–0100; Enzo Life Sciences, Inc., USA) diluted in buffer containing 0.3% Triton X-100 and 2.5% (v/v) normal goat serum at 4°C overnight. On the following day, the samples were washed three times (7 minutes each) with PBS (pH 7.4) and treated with a biotinylated goat anti-rabbit secondary antibody (1:200; Cat. #BA-1000–1.5; Vector Laboratories, USA) for 1 h at RT. Signals were detected by incubating sections with the avidin-biotin-peroxidase complex (Cat. #PK-6100; Vector Laboratories, USA) and subsequently with 3,3’-diaminobenzidine (DAB; Cat. #SK-4100; Vector Laboratories, USA) at RT. The brain sections were mounted onto glass slides (Cat. #4951PLUS-600621; New Erie Scientific LLC, USA), subsequently dehydrated in a series of graded ethanol (Cat. #A405P-4; Fisher Scientific, USA) and cleared in xylene (Cat. #X3S-4; Fisher Scientific, USA). The samples were covered with coverslips (2980245; Corning, USA) using Cytoseal-XYL xylene-based mounting medium (Cat. #Epredia^™^ 83124; Thermo Fisher Scientific, USA) and dried at RT. The samples were visualized under a light microscope (Cat. #TE360 Eclipse; Nikon, Japan) at 10× magnification. TH^+^ neurons in the substantia nigra were counted manually in brain sections following a procedure described previously(26). Six substantia nigra-containing sections were analyzed per mouse to determine the estimated number of total TH^+^ cells in the entire substantia nigra.

### Immunofluorescence analysis

Brain sections were washed with PBS (pH 7.4) and then incubated with blocking buffer containing 0.3% Triton X-100 and 5% (v/v) donkey serum (Cat. #D9663; Sigma–Aldrich, USA) in PBS (pH 7.4) to block nonspecific binding. The sections were then incubated with a rat monoclonal anti-GFAP antibody (1:500; Cat. #13–0300; Invitrogen, USA) prepared in buffer containing 0.3% Triton X-100 and 2.5% (v/v) donkey serum at 4°C overnight. The next day, the brain sections were washed three times in PBS and treated with a donkey anti-rat Alexa 488 secondary antibody (1:200; Cat. #A-21208; Molecular Probe, Invitrogen, USA). The brain sections were mounted onto glass slides and then covered with coverslips using Prolong Gold Antifade mountant (Cat. #P10144; Invitrogen, Thermo Fisher Scientific, USA). The samples were imaged under a confocal microscope (Nikon C2, Nikon, Japan) at 10× magnification. The quantification of the area occupied by GFAP^+^ cells normalized to the total area analyzed was performed by ImageJ (NIH) using four to six substantia nigra-containing sections per mouse(27).

### Caffeine treatment

Mice were given a dose of 20 mg/kg i.p. of caffeine (Cat. #CAS 58-08-2; Santa Cruz, USA) dissolved in 0.9% saline daily for two weeks(28, 29).

### Statistical analysis

Statistical analyses were conducted with GraphPad Prism version 9.0. Differences between two groups were analyzed using two-tailed Student’s t tests. Comparisons for more than two groups were performed using one-way ANOVA followed by Tukey’s multiple comparison post hoc test at an alpha level of 0.05. A difference was considered statistically significant if the P value was < 0.05.

## Results

### LPS does not cause the loss of dopaminergic neurons in either WT or LRRK2 G2019S-KI mice within two weeks

**Figure 1:**
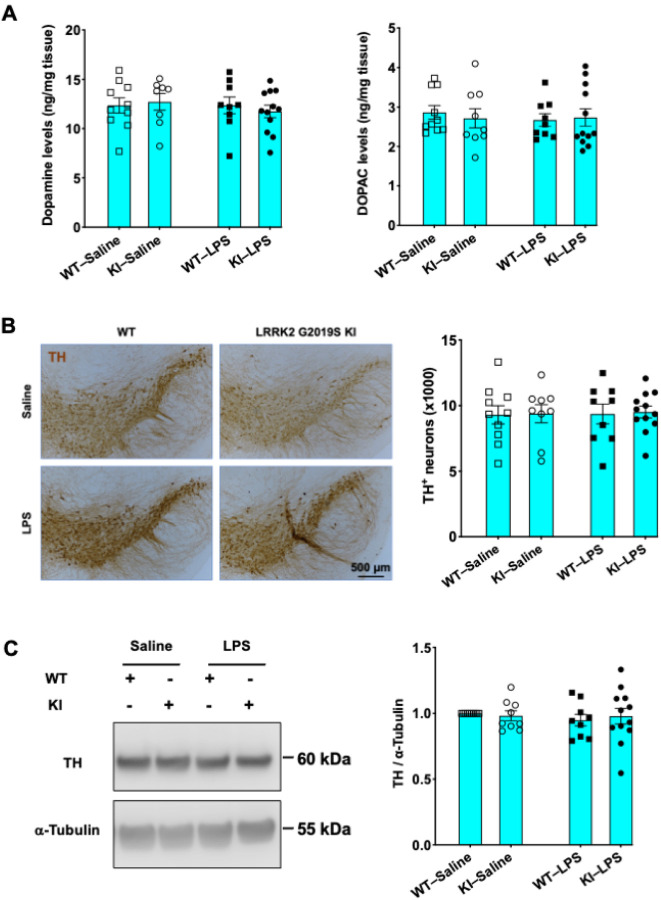
LPS administration does not cause dopaminergic neuron loss in WT or LRRK2 G2019S-KI mice within two weeks. (**A**) Striatal dopamine (left) and DOPAC (right) levels in WT and LRRK2 G2019S-KI mice injected with saline or LPS were analyzed by HPLC-ECD. (**B**) Representative images of brain sections containing TH^+^ neurons in the substantia nigra regions (left) and quantification of TH^+^ neurons in the substantia nigra area (right). Scale bar, 500 mm. (**C**) Striatal TH expression levels were measured by Western blot analysis (left). Densitometric quantifications are shown (right). a-Tubulin served as a loading control. *n* = 4 mice per group. For (A–C), one-way ANOVA followed by Tukey’s multiple comparison post hoc test was used. The data are presented as the means ± standard errors of the means (SEMs).

It has been reported that a single sublethal dose of LPS causes a delayed and progressive loss of dopaminergic neurons in WT mice. The degeneration of tyrosine hydroxylase (TH)^+^ neurons was not detected within four months but started to manifest seven months after LPS injection and progressed over time(20, 30). Notably, the neurodegenerative phenotype was accelerated in LRRK2 G2019S Tg mice, with dopaminergic neuronal death occurring as early as seven days after LPS challenge(21). Thus, we examined whether a similar exposure to LPS would cause any damage to the dopaminergic neurons of LRRK2 G2019S KI mice, even if not to those of WT mice. We administered a single dose of 5 mg/kg LPS to WT and LRRK2 G2019S KI mice and analyzed the brains of the mice collected on day 14 after LPS injection. We first measured the striatal levels of dopamine and its primary metabolite 3,4-dihydroxyphenylacetic acid (DOPAC) using HPLC-ECD. LPS injections did not cause a decrease in dopamine levels in mice of either genotype (Fig. 1A).

We next labeled TH^+^ neurons in serial brain sections containing substantia nigra samples using a TH antibody. There were no changes in the number of TH^+^ neurons two weeks after LPS injection in either the WT or LRRK2 G2019S-KI mice (Fig. 1B). We subsequently performed Western blot analysis to measure the TH levels in the striata and did not observe any alterations in the TH protein levels (Fig. 1C). Collectively, these results suggest that exposure to a single high dose of LPS did not lead to the loss of dopaminergic neurons in either WT or LRRK2 G2019S-KI mice within two weeks.

### LRRK2 G2019S-KI mice show incomplete recovery from LPS-induced weight loss

**Figure 2:**
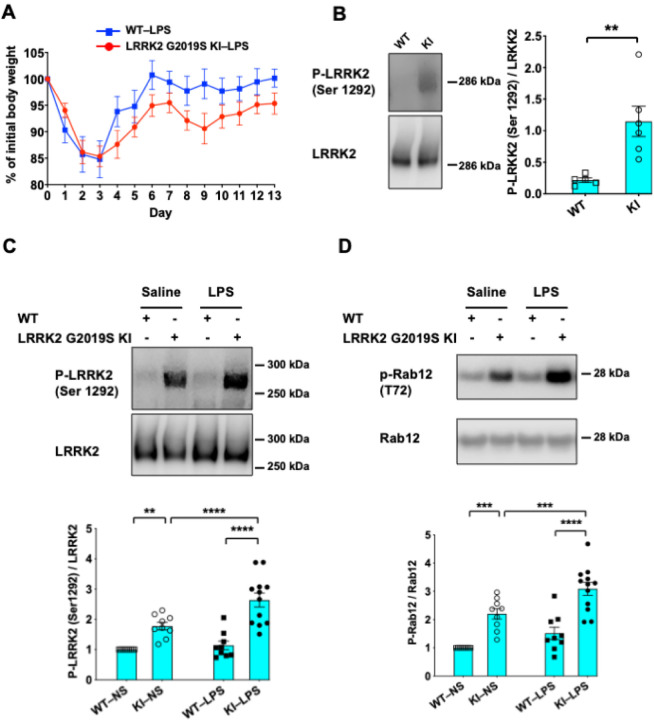
LPS-treated LRRK2 G2019S KI mice exhibit slower weight recovery and increased LRRK2 autophosphorylation at Ser1292. **(A)** Mice of the indicated genotypes were treated with a single dose of LPS (5 mg/kg, i.p.). Body weights were monitored daily for two weeks. The percentages of initial body weights are shown. The number of mice: WT, *N* = 8; LRRK2 G2019S KI, *N* = 11. One WT mouse died on day four post-LPS injection and was excluded from the study. (**B-D**) Striatal extracts from naive WT and LRRK2 G2019S-KI mice (**B**) and saline- or LPS-treated mice (**C, D**) were subjected to Western blot analysis. Representative blots of LRRK2, P-LRRK2 (Ser1292), and P-Rab12 (T72) (left) and corresponding quantification are shown (right). *n* = 5–8 mice per group. For (B), **P < 0.01; two-tailed Student’s *t test*; for (C), **P < 0.01; ****P < 0.0001; one-way ANOVA followed by Tukey’s multiple comparison post hoc test. The data are presented as the means ± SEMs.

Following LPS injection, mice of both genotypes displayed weight loss, which reached a nadir on day 3, consistent with the literature on the effects of LPS(31). Interestingly, LRRK2 G2019S KI mice regained weight more slowly than did WT mice (Fig. 2A). Specifically, the body weights of the WT mice returned to the baseline on day 6, whereas those of the mutant LRRK2 G2019S KI mice failed to recover to their baseline weights up to 13 days after LPS injection (Fig. 2A). These results suggest that the LRRK2 G2019S mutation leads to greater susceptibility to LPS in mice.

### LPS-treated LRRK2 G2019S KI mice display increased LRRK2 autophosphorylation at Ser1292 and phosphorylation of its substrate Rab12

The kinase activity of LRRK2 is central to its functions and toxicity(7, 32–34). Studies have suggested that the LRRK2 G2019S mutation confers an increase in the kinase activity of the protein. The autophosphorylation site Ser1292 has been proposed to be a direct readout of LRRK2 kinase activity(35, 36). Accordingly, we next checked the levels of phosphorylated Ser1292, P-LRRK2 (Ser1292), in lysates from mouse striata, the region most affected in PD (37) and with the highest expression levels of LRRK2 in the brain (5). Western blot analyses revealed that P-LRRK2 (Ser 1292) was barely detectable in the striatal extracts of WT mice but strongly expressed in those of LRRK2 G2019S KI mice (Fig. 2B). These results suggest that LRRK2 kinase activity is enhanced in LRRK2 G2019S KI mice.

To determine whether the sensitivity of LRRK2 G2019S KI mice to LPS is associated with enhanced kinase activity, we next measured P-LRRK2 (Ser 1292) levels in mice treated with LPS via Western blot analyses. LPS exposure further increased P-LRRK2 (Ser 1292) expression in the LRRK2 G2019S-KI mutant mice (Fig. 2C). Similarly, we found that the expression level of the LRRK2 substrate Rab 12 (38) was greater in naive KI mice than in WT mice, which was further induced by LPS treatment (Fig. 2D). This result suggested that LRRK2 is overactivated in LRRK2 G2019S KI mice challenged with LPS. This overactivation could underlie the susceptibility of the mutant LRRK2 G2019S KI mice to LPS.

### LRRK2 G2019S-KI mice exhibit greater LPS-induced increases in the levels of the astrocytic marker GFAP

**Figure 3:**
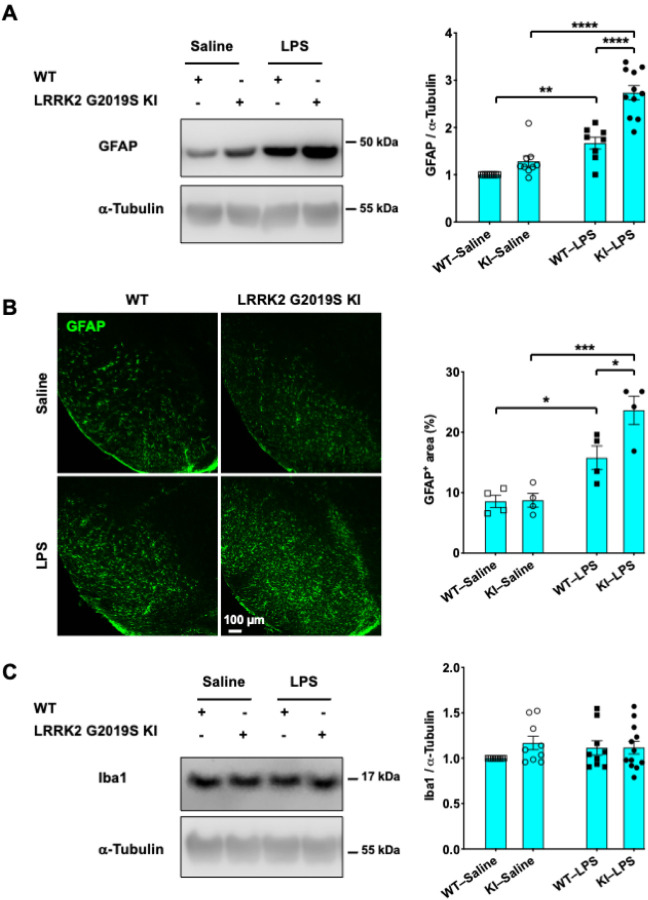
LRRK2 G2019S-KI mice exhibit increased LPS-induced astrocyte activation. **(A)** GFAP expression levels in striatal extracts were measured by Western blot analysis (left). Densitometric quantifications are shown (right). (**B**) Substantia nigra-containing brain sections were immunostained with an anti-GFAP antibody (green). Representative confocal images (left) and quantification of the GFAP^+^ area normalized to the total area are shown (right). Scale bar, 100 mm. (**C**) Iba1 expression levels in striatal extracts were measured by Western blot analysis (left). Densitometric quantifications are shown (right). a-Tubulin was used as a loading *control*. *n* = 3–4 mice per group. For (A−C), *P < 0.05; ***P < 0.001; one-way ANOVA followed by Tukey’s multiple comparison post hoc test. The data are presented as the means ± SEMs.

LRRK2 is constitutively expressed in astrocytes (13) and microglia(14), suggesting the involvement of LRRK2 in neuroinflammation and PD. Thus, we next investigated inflammatory responses in the brain areas affected by PD in WT and LRRK2 G2019S-KI mice challenged with LPS. We first subjected tissue lysates from the striata of mice receiving either saline or LPS to Western blot analyses. LPS significantly increased the expression of GFAP, a marker of activated astrocytes(39). Interestingly, we noticed an effect of genotype, as LPS-challenged LRRK2 G2019S KI mice displayed greater levels of GFAP than WT mice receiving the same treatment (Fig. 3A). Next, we performed GFAP staining of brain sections containing substantia nigra samples. We also found that LPS treatment increased the expression of GFAP to a greater extent in LRRK2 G2019S KI mice than in WT mice (Fig. 3B). There were no changes in the levels of the activated microglial marker Iba1 in striatal tissue in any of the groups, regardless of treatment or mouse genotype (Fig. 3C). This finding suggested that microglia returned to baseline levels on day 14 after LPS exposure. Collectively, these results suggest that LPS-induced astrocyte activation is exacerbated in LRRK2 G2019S KI mice two weeks after LPS exposure.

### Caffeine attenuated LPS-induced astrocyte activation in LRRK2 G2019S KI mice but not LPS-induced increases in the phosphorylation of LRRK2 or its substrate Rab12

**Figure 4:**
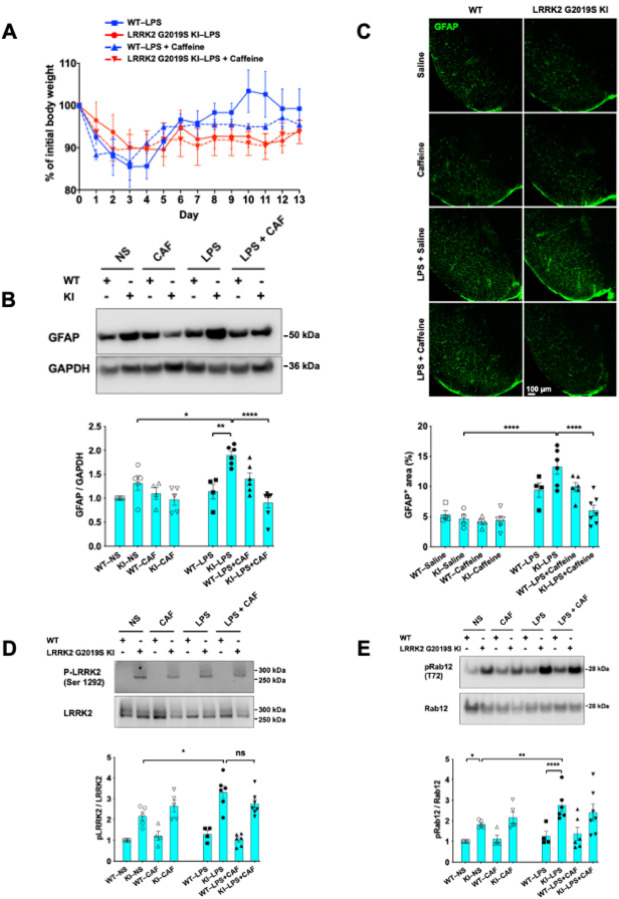
Caffeine ameliorates LPS-induced astrocyte activation in LRRK2 G2019S KI mice. **(A)** Mice of both genotypes were treated with a single dose of LPS (5 mg/kg, i.p.) or caffeine (daily; 20 mg/kg, i.p.). Body weights were monitored daily for two weeks. The percentages of initial body weights are shown. The number of mice was as follows: WT–LPS, N = 4; LRRK2 G2019S–LPS, *N* = 6; WT–LPS + caffeine, *N* = 6; and LRRK2 G2019S KI–LPS + caffeine, *N* = 7. One WT mouse in the LPS-treated group died on day two post-LPS injection and was excluded from the study. (**B**) Striatal extracts were subjected to Western blot analysis to measure GFAP expression levels (top). The corresponding quantifications are shown (bottom). (**C**) Brain sections containing the substantia nigra were immunostained with an anti-GFAP antibody (green). Representative confocal images (top) and quantification of the GFAP^+^ area normalized to the total area are shown (bottom). Scale bar, 100 mm. (**D-E**) Striatal extracts were subjected to Western blot analysis to measure P-LRRK2 (Ser1292) (**D**) and P-Rab12 (T72) (**E**) expression levels (left). The corresponding quantifications are shown (right). *n* = 4–7 mice per group. For (B-E), *P < 0.05; **P < 0.01; ****P < 0.0001, n.s.: nonsignificant; one-way ANOVA followed by Tukey’s multiple comparison post hoc test. The data are presented as the means ± SEMs.

We next investigated the effects of caffeine on LPS-treated WT and LRRRK2 G2019S-KI mice. Mice of each genotype challenged with LPS were given a daily dose of 20 mg/kg caffeine for 14 days. The dose of caffeine is optimized for CNS effects in mice and is relevant to human caffeine exposure(40, 41). Compared with WT mice, LRRK2 G2019S-KI mice showed incomplete recovery of body weight gain after LPS injection (Fig. 4A). After the recovery of some weight loss, initial caffeine administration appeared to diminish weight recovery in LPS-treated WT mice (Fig. 4A). In contrast, LRRK2 G2019S-KI mice regained minimal weight with or without caffeine administration (Fig. 4A). Interestingly, caffeine administration reduced GFAP levels in both striata (Fig. 4B) and substantia nigra (Fig. 4C) only in LPS-treated LRRK2 G2019S KI mice but not in LPS-challenged WT mice. However, we did not observe any effects of caffeine treatment on the phosphorylation of LRRK2 or its substrate Rab12 induced by LPS in LRRK2 G2019S KI mice (**Fig. 5**).

## Discussion

Mutations in the *LRRK2* gene are among the most common genetic causes of PD, yet the clinical features of LRRK2 PD are largely indistinguishable from those of sporadic PD(6, 42). LRRK2 also reportedly plays a role in idiopathic PD with postmortem brain tissue from patients with idiopathic PD showing enhanced LRRK2 kinase activity(8). Understanding LRRK2 PD should therefore provide more insights into the underlying mechanisms and help create new therapeutic opportunities for idiopathic PD(42). However, there is no murine LRRK2 PD model that fully recapitulates human PD(43).

The G2019S mutation is one of the most common mutations of LRRK2 in PD and has activating and gain-of-function effects on LRRK2 kinase activity(6). Studies using LRRK2 G2019S Tg mice have shown some PD-related phenotypes. These include loss of dopaminergic neurons, disruption of dopamine homeostasis, which is accompanied by dopamine-dependent behavioral deficits, and α-synucleinopathies(44–46). A degenerative phenotype in the substantia nigra of these mice is typically observed in aged mice at approximately 15–20 months of age(44, 46). In 1-methyl-4-phenyl-1,2,3,6-tetrahydropyridine (MPTP)-induced models of PD, LRRK2 G2019S Tg mice were more susceptible to MPTP-mediated neurotoxicity(47, 48). Although LRRK2 G2019S Tg mice can display many of the cardinal features of PD, these models bear several key caveats due to overexpression artifacts or interspecies differences(43). LRRK2 G2019S KI models have been developed to overcome potentially confounding insertional effects of the Tg models(22, 49). However, LRRK2 G2019S KI mouse models have failed to exhibit dopaminergic neuron degeneration or α-synuclein pathology(22, 49, 50). This is probably due to the incomplete penetrance of the mutation(5). These findings suggest the involvement of other environmental or genetic factors in establishing PD models in LRRK2 G2019S KI mice.

Neuroinflammation is increasingly recognized as an essential process involved in PD pathogenesis(51). Injection of a sublethal dose of LPS reportedly resulted in the loss of dopaminergic neurons in LRRK2 G2019S Tg mice as early as seven days after treatment(21). However, we did not observe a similar phenotype in our LRRK2 G2019S KI mice receiving the same dose of LPS two weeks after LPS exposure. Although it is challenging to compare these two studies, we speculate that the reason for the difference might be the context of the G2019S mutation in transgenic versus endogenous LRRK2. The toxicity of LPS to dopaminergic neurons might have been intensified in the LRRK2 G2019S Tg mice utilized by Kozina and colleagues, which displayed a high expression level of LRRK2 G2019S(21). In the scope of our current study, we have not yet established a mouse model of inflammation-associated LRRK2 PD. Previous studies reported that the LPS paradigm in WT mice maintained for 10 months after inflammatory exposure was sufficient to cause dopaminergic neuronal death(20, 30). With astrocyte activation being exacerbated in LRRK2 G2019S KI mice, we anticipate that when we extend the duration of LPS exposure, LRRK2 G2019S KI mutant mice will potentially exhibit an accelerated neurodegenerative phenotype. This hypothesis merits further investigation.

The roles of LRRK2 in microglia have been extensively investigated(14, 52–54), but little is known about the functions of LRRK2 in astrocytes(55–57). Under physiological conditions, astrocytes are essential for brain homeostasis because they provide neurotrophic factors to support neurons and promote synapse formation and plasticity(58). However, astrocytes undergo sequential phenotypic changes called “reactive astrocytosis” in response to brain injuries and diseases. Reactive astrocytes are present and drive neuronal death in many neurodegenerative disorders, including PD(19, 39). Recent studies have reported alterations in astrocytes in *LRRK2* PD patients. These include morphological and metabolic alterations, which are recognized as pathological features of PD(59, 60). It has been reported that induced pluripotent stem cell (iPSC)-derived astrocytes from PD patients carrying the LRRK2 G2019S mutation exhibit many PD features, such as increased expression of α-synuclein, thereby increasing the responsiveness of these cells to inflammatory stimuli(59). In another study, astrocytes generated from the iPSCs of PD patients with LRRK2 G2019S mutations exhibited irregular mitochondrial morphology, decreased mitochondrial activity and ATP production, and increased reactive oxygen species (ROS) production(60). These astrocytes thus fail to support neurons homeostatically and may have contributed to dopaminergic neurodegeneration.]

In the model of neuroinflammation triggered by LPS, we observed an astrocyte activation-potentiating phenotype in LRRK2 G2019S KI mice. Further studies are needed to understand the toxicity of activated astrocytes to neurons and the underlying mechanisms involved. In addition, with a greater degree of astrocyte activation, LRRK2 G2019S KI mice could serve as a mouse model to study the anti-inflammatory effects of small molecules and their specificity for LRRK2.

Caffeine is an adenosine A_2A_ receptor antagonist and the most widely consumed psychoactive substance(61). Epidemiological and metabolomic studies have shown that higher coffee and caffeine intake is associated with a reduced risk of PD(62, 63), and the serum levels of caffeine and its metabolites are lower in idiopathic PD patients(61, 63). Preclinical models also support the neuroprotective effects of caffeine in PD(40, 64, 65). In a metabolomic profiling study carried out to identify markers of resistance to developing PD among *LRRK2* mutation carriers, our group identified caffeine and its related analytes as potential modulators(23). We observed evidence of protection by caffeine against LPS-potentiated astrocyte activation in mice bearing the pathogenic mutation G2019S of LRRK2 at a dosage producing concentrations achieved with typical human consumption(40, 41). The effects of caffeine in the CNS, including its psychostimulant and neuroprotective effects, are mediated by its antagonistic effects on adenosine receptors(66), most prominently on A_2A_ receptors(41). LRRK2 expression is enriched in the striatum(67, 68), the brain area that is laden with A_2A_ receptors(69), and is also known to play a role in developing striatal circuits(70). Thus, the involvement of LRRK2 in striatal neuroplasticity may underlie the potentiation of the neuroprotective effects of caffeine in the context of pathogenic LRRK2 mutations. Interestingly, caffeine’s effects appear to be specific to LRRK2 G2019S KI mice in our current study. The enhanced kinase activity of LRRK2 appears to underlie the toxicity of the protein in the model. However, we did not find any evidence that caffeine ameliorates the phosphorylation of the protein at its phosphorylation site or its substrate, which is a readout of its kinase activity(7, 32, 33). Further studies will be needed to unravel the molecular networks that are potentially altered by the G2019S mutation of LRRK2 and how they are modulated by caffeine treatment. As caffeine specifically attenuates a pathogenic phenotype in LRRK2 G2019S KI mice, the development of this relatively low-risk dietary and pharmacological agent as a candidate therapeutic for PD patients with *LRRK2* mutations or as a preventive strategy for at-risk *LRRK2* mutation carriers to reduce the penetrance of PD is possible.

In summary, our findings revealed that in a model of inflammation triggered by LPS, LRRK2 G2019S KI mice displayed increased LRRK2 kinase activity. The enhanced kinase activity of LRRK2 in LPS-challenged LRRK2 G2019S KI mice was likely accompanied by delayed weight recovery and increased astrogliosis, which was ameliorated by caffeine administration. Our findings thus add to the increasing body of evidence that the kinase activity of LRRK2 underlies its toxicity in PD pathogenesis and support the therapeutic potential of caffeine in LRRK2 PD.

## Conclusion

Our findings suggest that LRRK2 G2019S KI mice exhibit heightened neuroinflammatory responses and impaired recovery from a peripheral challenge, potentially mediated by astrocyte activation. While caffeine appears to specifically modulate astrocytic responses in LRRK2 G2019S KI mice, further investigation is needed to elucidate the underlying mechanisms and its potential therapeutic implications.

## Data Availability

All relevant data are contained within this article.

## Abbreviations

PD: Parkinson’s disease
LRRK2: Leucine-rich repeat kinase 2
KI: Knock-in
WT: Wild-type
LPS: Lipopolysaccharide
CNS: Central nervous system
TH: Tyrosine hydroxylase
GFAP: Glial brillary acidic protein
Iba1: Ionized calcium-binding adaptor molecule 1
HPLC: High-performance liquid chromatography
ECD: Electrochemical detection
DOPAC: 3 4-dihydroxyphenylacetic acid
PFA: Paraformaldehyde
PBS: Phosphate buffered saline
SEM: Standard error of the mean
